# Seven-year clinical follow-up of premanifest carriers of Huntington's disease

**DOI:** 10.1371/currents.RRN1288

**Published:** 2011-11-30

**Authors:** Ellen Hart, Huub Middelkoop, Caroline K Jurgens, Marie-Noëlle W Witjes-Ané, Raymund A.C. Roos

**Affiliations:** ^*^Neuropsychologist and researcher, Department of Neurology, Leiden University Medical Centre, P.O. Box 9600, 2300 RC Leiden, The Netherlands; ^†^Leiden University Medical Center; ^‡^Leiden University Medical Center, Leiden; Bronovo hospital, The Hague, The Netherlands and ^¶^LUMC, Leiden, The Netherlands

## Abstract

Detecting subtle clinical abnormalities in the ‘premanifest’ phase of Huntington’s disease (HD) is of importance in the development of instruments to monitor early therapeutic intervention trials. The current study examined changes in motor function, cognition and behaviour over a period of seven years in premanifest carriers of the HD gene mutation. Twenty-nine carriers without unequivocal motor signs of HD and 43 non-carrier controls were prospectively examined four times. The assessments consisted of the Unified Huntington’s Disease Rating Scale (UHDRS) and an extensive neuropsychological test battery addressing global cognitive function, memory, language and executive function. Rate of Change (RoC) analysis was performed to measure longitudinal differences between carriers and non-carriers. Carriers performed consistently worse on executive function (Symbol Digit Modalities Test (SDMT), Stroop, Trail Making Test (TMT) and WAIS-R arithmetic). Over the years, carriers showed a decline in memory and concentration function (Wechsler Memory Scale (WMS)) and in motor function (UHDRS motor scale). Changes over time could be particularly ascribed to carriers converting to manifest HD. These results demonstrate that standardized motor assessments and objective memory and concentration tasks are sensitive to change over a period of 7 years, specifically in carriers converting to manifest HD. Executive tasks also showed subtle cognitive abnormalities in premanifest HD, but a decline over time could not be demonstrated.

**Introduction**

Huntington’s disease (HD) is a hereditary neurodegenerative disease that becomes manifest in midlife and is characterised by motor disturbances, cognitive decline and behavioural dysfunction. HD is caused by the abnormal expansion of a trinucleotide (CAG) repeat in the gene for the protein huntingtin [1]. The genetic defect leads to cerebral cell death, especially in the basal ganglia. Although the clinical diagnosis of HD is based on the first appearance of motor signs, a positive family history and confirmation by DNA-testing, subtle changes in motor function, cognition and behaviour are known to precede manifest disease. Detecting these very early changes is of importance in the development of instruments to monitor early therapeutic intervention trials and to obtain more insight into the phase of clinical disease onset. 

    Cross-sectional reports showed that carriers of the HD gene mutation without manifest motor signs (further labelled as carriers) perform significantly worse than non-carriers on certain motor scores, neuropsychological tests and behavioural assessments [2] [3] [4] [5] [6] [7] [8] [9] [10] [11]. Furthermore, some studies showed relationships between fewer years to estimated onset of diagnosable clinical disease and worse motor and cognitive function [2] [7] [12] [13] [14].

    Longitudinal follow-up is however necessary to really understand the pattern of evolving motor, cognitive and behavioural abnormalities in the phase of HD clinical onset. First of all, to find out if clinical markers that are sensitive in detecting premanifest abnormalities show a decline over time and might be suitable as outcome measures in future therapeutic trials. Secondly, to monitor whether carriers, converting to manifest HD, display specific clinical changes.       To date, several longitudinal studies have been undertaken. The duration of follow-up however has been brief in the vast majority of longitudinal studies and results show discrepancies. In accordance with other studies we failed to demonstrate clinical markers for premanifest HD in our previous 3-year follow-up study [15] [16] [17]. Others did detect a significant decline over time in motor function, executive function, attention and memory [18] [19] [20] [21]. The longest follow-up study to date (10 years) demonstrated that the most rapid decline on motor and cognitive domains was found in individuals approaching clinical disease onset [13].       With the present observational study we aim to give more insight in the clinical onset phase of the disease as the number of reports on clinical decline in premanifest HD using long-term follow-up with a comprehensive assessment battery is limited. The objective was to follow a premanifest HD carrier group for 7 years in order to determine if our assessment battery detects subtle clinical changes preceding diagnosable clinical HD. Furthermore, we examined whether the rate of decline on motor, cognitive and behavioural measures could be related to estimated proximity to diagnosable clinical disease.    

**Methods**

***Participants***

In the original study, 134 participants were included (46 premanifest carriers, 88 non-carriers). They were referred to the Leiden University Medical Centre (LUMC) Department of Clinical Genetics, for predictive testing, and consented to participate in a three-year clinical follow-up study [17]. Non-carriers were at-risk individuals who, after predictive testing, proved to have a CAG-repeat lenght of fewer than 27. Carriers were considered to be premanifest in the absence of unequivocal motor signs on the Unified Huntington’s Disease Rating Scale (UHDRS) [22]. A neurologist who was blind to genetic status and trained in the administration of the UHDRS performed the motor examination and filled in a score ranging from 0-3 (0= normal, 1= minor soft signs, 2= probable HD, 3= unquestionable HD). Carriers with a rating of 3 at baseline were diagnosed with HD and excluded from the study. One hundred six participants (33 carriers, 73 non-carriers) attended all follow-ups in the three-year period. Seven years after the start of the original study these participants were invited to take part in an additional follow-up. Twenty-nine carriers and 44 non-carriers consented to continue follow-up. Four carriers and 29 non-carriers did not re-enter the study after the original three year follow-up. One non-carrier was excluded for analyses since he had a minor stroke. This seven year follow-up study, therefore, reports on 29 carriers who were premanifest at baseline, and 43 non-carriers who served as controls. Carriers who where rated as unquestionable HD on the UHDRS after seven years were considered converters to manifest HD. The study was approved by the local Medical Ethical Committee. Written informed consent was obtained from all participants.     

***Procedure***

All participants were evaluated with the UHDRS, including motor and behavioural assessment, and an extensive set of neuropsychological tests covering global cognitive function, memory, language and executive function (Tables 2 and 3) [17] [22]. The number of estimated years to clinical diagnosis (EYTD) was calculated using a CAG- and age-based predictive model designed by Langbehn et al. [23].    

*Motor assessment*

The UHDRS motor rating was filled in by a neurologist (range 0-3). A Total Motor Score (TMS) was calculated by summing all motor items of the UHDRS [17]. Analysis of TMS subscales was restricted to eye movement, voluntary movement and chorea [22].   

*Neuropsychological assessment*

Neuropsychological tests included Mini Mental State Examination (MMSE) [24]; Wechsler Adult Intelligence Scale-Revised (WAIS-R) [25] subtests Information, Digit span, Arithmetic, Picture arrangement, Block design; Wechsler Memory Scale (WMS) [26];Verbal fluency (FAS) [27]; Boston naming test [28]; Symbol Digit Modalities Test (SDMT) [29]; Trail Making Test, consisting of a simple (TMT-A) and a more complex (TMT-B) version [30];Stroop colour-word test [31]. Reaction time measures were both derived from a simple reaction time paradigm and a complex ‘choice’ reaction time paradigm (go/no go paradigm) [32]. A psychologist administered the cognitive tests [17].  

*Behavioural assessment*

Behavioural and mood complaints were limited to the total behavioural score (TBS) that was obtained by adding the products of the frequency and severity for each item from the behavioural assessment of the UHDRS, administered by a psychologist [11]. Analysis of individual items from the behavioural assessment were restricted to four frequently reported neuropsychiatric symptoms; sadness, anxiety, aggression and irritability [33].   

*Statistical analysis*

SPSS for Windows (release 16.0) was used for data analysis. Cross-sectional group differences at baseline and after 7 years were analysed with parametric or non-parametric tests when appropriate. Clinical group comparisons were corrected for age at assessment using ANCOVA. For longitudinal analyses we used the method reported by Solomon et al. (2008) since it accounts for slight differences in follow-up period (Solomon et al., 2008). For each clinical score the change over the 7-year period was calculated for each participant. This Rate of Change (further labelled as RoC) was computed as follows: RoC= (score_2_- score_1_)/ (age_2_-age_1_).  Differences in RoC between carriers and non-carriers were analysed using ANCOVA with correction for age at baseline. To assess the relationship between EYTD and RoC, Pearson correlation coefficients were calculated. The level of statistical significance was set at p≤ 0.01. A more liberal level of significance of 0.01<p £ 0.05 was also reported to be of marginal interest to maximize any opportunity of finding trends towards group differences. Effect sizes are displayed as partial eta squared values (_p_h^2^), eta squared values (h^2^) and r-values.   

**Results**  

*Group characteristics at study entry*

The mean time interval between baseline and seven-year follow-up was 7.3 years (range 6.3-8.5 years). The group characteristics are described in Table 1. Carriers were younger than non-carriers (p= .015,h^2^= .082). No group differences emerged for gender and education.  

Table 1. Characteristics of carriers and non-carriers at baseline 

**Table:** 

	Carriers n=29	Non-carriers n=43	P
Male/female^a^	11/18	18/25	ns
Age (years)	37.9 (9.1)	43.8 (10.4)	.015*
Education (years)	12.0 (2.9)	11.9 (2.8)	ns
CAG repeat length^b^	42.9 (39-49)	20.7 (16-30)	
Estimated years to clinical diagnosis (EYTD)^b^	15.6 (4.3-36.4)	-	

Values in the table are means with SD or range^b^ between parentheses. Independent t-tests were used except where Pearson c^2^-test was used ^a^. *p≤ .05, **p ≤.01.

*Cross-sectional Results*

Carriers did not differ from non-carriers with respect to the UHDRS motor rating at baseline (p= .99, r= -0.002) or after seven years (p= .12,r= -0.192) (Table 2). Five carriers (17%) converted to unquestionable HD on the UHDRS during follow-up. Two converters were rated as normal at baseline. Three converters were rated as probable HD at baseline. One carrier who was rated as probable HD at baseline was rated as normal after 7 years. Three non-carriers were rated as probable HD at baseline and five after 7 years. None of the non-carriers was rated as unquestionable HD. From the three carriers where motor assessment was missing at baseline, two were rated as normal after 7 years and one carrier showed minor soft signs.  

Mean clinical scores are displayed in Table 3. At baseline, carriers showed more complaints in aggression than non-carriers (p= .024,_ p_h^2^= .073). They also performed worse on the WAIS-R arithmetic subsection (p= .031,_ p_h^2^= .065), SDMT (p=.002,_ p_h^2^= .128), TMT-A (p= .006,_ p_h^2^= .104), TMT-B (p= .024,_ p_h^2^= .072), Stroop word (p= .008,_ p_h^2^= .097) and Stroop interference (p=.01,_ p_h^2^= .089). After seven years carriers additionally showed more motor abnormalities compared to non-carriers on the UHDRS TMS (p= .012,_ p_h^2^= .096), and on the UHDRS eye movement (p= .026,_ p_h^2^= .076), voluntary movement (p= .029,_ p_h^2^= .073) and chorea subsection (p= .005,_ p_h^2^= .118). Also, worse scores on the MMSE (p= .015,_ p_h^2^= .083), WMS memory quotient (p= .001,h^2^= .147), WMS concentration (p= .001,_ p_h^2^= .161), WMS logical memory (p= .025,_ p_h^2^= .07) WMS visual reproduction (p= .042,_ p_h^2^= .059) and Stroop colour (p= .009,_ p_h^2^= .097) emerged in carriers. Cross-sectional differences at baseline on aggression and Stroop word could not be demonstrated after seven years.   

Without the five carriers who converted to manifest HD, baseline differences remained, except for TMT-B (p= .144,_p_h^2^= .033). In the analyses after seven years, differences remained only on UHDRS TMS (p= .04,_ p_h^2^= .071) WMS memory quotient (p=.007,h^2^= .105), WMS concentration (p=.006,_ p_h^2^= .114) and Stroop colour (p= .027, _p_h^2^= .075). 

Table 2. Frequencies of UHDRS motor ratings in carriers and non-carriers at baseline and after 7 years

**Table:** 

Carriers Non-carriers (n= 29) (n= 43) UHDRS rating Baseline Seven years Baseline Seven years				
Normal	19	15	29	24
Minor soft signs	3	5	9	8
Probable HD	4	4	3	5
Unquestionable HD	-	5	-	-

Values are expressed as number. UHDRS= Unified Huntington’s Disease Rating Scale. Out of 29 carriers 3 motor assessments were missing at baseline. Out of 43 non-carriers two motor assessments were missing at baseline and six at follow-up.

Table 3. Mean (SD) performances on motor, behavioural and cognitive assessment at baseline and seven-year follow up in carriers and non-carriers  

**Table:** 

**Baseline** Carriers Non-carriers P _p_h^2^					**Seven years ** Carriers Non-carriers P _p_h^2^				
**Motor** ^b^									
UHDRS TMS	6.9 (7.8)	6.3 (5.7)	.124	.037		8.0 (15.5)	2.8 (2.7)	.012*	.096
Eye movement	2.2 (3.3)	1.9 (2.8)	.113	.039		1.8 (4.2)	0.9 (1.3)	.026*	.076
Voluntary movement	2.1 (2.1)	1.9 (2.0)	.135	.035		2.1 (3.9)	0.9 (1.3)	.029*	.073
Chorea	1.2 (2.5)	0.9 (2.1)	.363	.013		2.6 (5.2)	0.5 (0.8)	.005**	.118
**Behaviour** ^b^									
UHDRS TBS	9.0 (13.4)	5.2 (7.2)	.484	.007		9.6 (13.1)	8.1 (12.6)	.786	.001
Sadness	1.9 (2.8)	1.3 (2.2)	.989	.00		2.0 (3.2)	2.0 (3.0)	.436	.009
Anxiety	1.3 (2.7)	1.0 (1.9)	.702	.002		1.8 (2.8)	1.3 (2.5)	.873	.00
Aggression	1.5 (3.0)	0.2 (0.8)	.024*	.073		1.6 (3.0)	0.8 (2.3)	.558	.005
Irritability	1.4 (2.8)	1.0 (2.1)	.696	.002		1.6 (2.7)	1.7 (2.7)	.383	.011
**Neuropsychology**									
MMSE, total score	28.2 (1.1)	28.6 (1.3)	.248	.019		28.3 (1.3)	29.0 (1.2)	.015*	.083
WAIS-R Information	10.4 (3.0)	10.8 (1.8)	.932	.00		10.6 (2.8)	10.9 (2.0)	.899	.00
Digit span	8.1 (2.6)	8.9 (2.2)	.076	.045		8.4 (3.0)	9.3 (2.1)	.078	.044
Arithmetic	10.6 (3.3)	12.3 (2.5)	.031*	.065		10.6 (3.4)	12.4 (2.6)	.023*	.073
Picture arrangement	8.8 (2.5)	9.2 (2.6)	.217	.022		9.3 (2.7)	9.2 (3.1)	.347	.013
Block design	11.8 (3.0)	11.7 (2.9)	.591	.004		11.6 (3.3)	11.9 (2.9)	.118	.036
WMS, memory quotient^a^	115.2 (15.6)	122.1 (14.7)	.062	.05		113.8 (19.0)	127.8 (15.3)	.001**	.147
Concentration	7.7 (1.8)	8.4 (1.2)	.107	.037		6.8 (2.1)	8.4 (1.2)	.001**	.161
Logical memory	9.4 (3.1)	10.1 (3.2)	.237	.02		8.2 (3.3)	10.0 (3.6)	.025*	.07
Visual reproduction	11.1 (2.3)	11.2 (2.2)	.686	.002		10.2 (3.3)	11.2 (2.2)	.042*	.059
Associative learning	18.0 (2.2)	18.0 (2.6)	.719	.002		17.2 (2.9)	17.5 (3.0)	.600	.004
Verbal fluency (FAS)	33.7 (9.4)	34.2 (11.5)	.869	.000		34.8 (14.0)	37.4 (12.0)	.387	.011
Boston naming test	26.6 (2.6)	26.1 (4.6)	.712	.002		27.1 (2.5)	27.7 (2.0)	.308	.015
SDMT, total score	48.2 (10.8)	53.2 (10.1)	.002**	.128		53.3 (15.8)	58.7 (10.5)	.006**	.103
TMT-A, seconds^b^	38.3 (14.8)	31.2 (9.4)	.006**	.104		33.0 (17.6)	28.4 (9.4)	.035*	.063
TMT-B, seconds^b^	59.6 (22.9)	51.4 (17.4)	.024*	.072		66.1 (47.1)	52.1 (23.2)	.017*	.079
Stroop colour	74.0 (11.7)	77.4 (10.8)	.054	.053		71.5 (14.6)	77.6 (13.3)	.009**	.097
Stroop word	95.5 (16.5)	103.4 (14.4)	.008**	.097		96.1 (20.4)	101.2 (15.7)	.086	.043
Stroop interference	42.1 (10.4)	45.2 (8.5)	.01**	.089		41.5 (9.8)	45.6 (8.8)	.001**	.150
Reaction time simple, milliseconds^b^	428.5 (70.5)	422.2 (63.7)	.298	.017		484.6 (113.5)	455.8 (80.2)	.135	.036
Reaction time complex, milliseconds^b^	568.1 (84.8)	552.7 (85.0)	.146	.033		640.8 (127.6)	615.0 (106.2)	.112	.041

Mean scores (SD). Raw scores are displayed except for WMS memory quotient, which is calculated with a correction for age. ANCOVA corrected for age except for^a^. *p≤ .05, **p ≤.01. Effect sizes are displayed as partial eta squared  (_p_h^2^) values. Higher scores correspond with worse performance^b^. UHDRS= Unified Huntington’s Disease Rating Scale. TMS= Total Motor Score. TBS= Total Behavioural Score. MMSE= Mini Mental State Examination, WAIS-R= Wechsler Adult Intelligence Scale-Revised. WMS= Wechsler Memory Scale, SDMT= Symbol Digit Modalities Test (number correct), TMT= Trail Making Test (sec), Reaction time simple= Reaction time single stimulus conditions (milliseconds), Reaction time complex= Reaction time complex conditions (milliseconds).  

*Longitudinal Results *The Rates of Change (RoC) on the motor, behavioural and cognitive tests are displayed in Table 4. Carriers demonstrated a greater rate of decline compared to non-carriers on the UHDRS TMS (p= 0.024,_ p_h^2^= .083) and chorea subsection (p= .008,_ p_h^2^= .114), the WMS memory quotient (p= .015,_ p_h^2^= .084), WMS concentration (p= .007,_ p_h^2^= .101) and WMS visual reproduction (p= .045,_ p_h^2^= .057). Without the carriers who converted to manifest HD, the only remaining significant decline in carriers compared to non-carriers was on WMS concentration (p= .041,_ p_h^2^= .063).  

Table 4. Mean (SD) Rate of Change (RoC) in carriers and non-carriers between baseline and 7-year follow-up

**Table:** 

Carriers Non-carriers (n= 29) (n=43) RoC RoC P _p_h^2^				
**Motor** ^b^				
UHDRS TMS	0.217 (1.440)	-0.475 (0.639)	.024*	.083
Eye movement	-0.037 (0.401)	-0.141 (0.294)	.431	.011
Voluntary movement	0.012 (0.375)	-0.149 (0.252)	.103	.045
Chorea	0.235 (0.523)	-0.052 (0.328)	.008**	.114
**Behavioural** ^ b^				
UHDRS TBS	0.164 (2.178)	0.380 (1.485)	.634	.003
Sadness	0.025 (0.581)	0.093 (0.313)	.634	.003
Anxiety	0.079 (0.556)	0.043 (0.326)	.554	.005
Aggression	0.019 (0.457)	0.084 (0.321)	.388	.011
Irritability	0.039 (0.493)	0.091 (0.375)	.401	.010
**Neuropsychological**				
MMSE, total score	0.012 (0.213)	0.057 (0.200)	.319	.014
WAIS-R Information	0.030 (0.236)	0.007 (0.129)	.706	.002
Digit span	0.048 (0.260)	0.057 (0.225)	.994	.000
Arithmetic	-0.006 (0.321)	0.016 (0.296)	.728	.002
Picture arrangement	0.067 (0.255)	0.004 (0.314)	.745	.002
Block design	-0.028 (0.314)	0.035 (0.228)	.156	.029
WMS, memory quotient	-0.265 (1.813)	0.760 (1.593)	.015*	.084
Concentration	-0.134 (0.236)	0.004 (0.148)	.007**	.101
Logical memory	-0.170 (0.369)	-0.015 (0.298)	.065	.049
Visual reproduction	-0.129 (0.327)	0.008 (0.313)	.045*	.057
Associative learning	-0.119 (0.330)	-0.046 (0.368)	.656	.003
Verbal fluency (FAS)	0.187 (1.181)	0.428 (1.131)	.174	.027
Boston naming test	0.074 (0.193)	0.211 (0.622)	.351	.013
SDMT, total score	0.709 (1.440)	0.723 (0.780)	.827	.001
TMT-A, seconds^b^	-0.781 (2.073)	-0.375 (1.105)	.446	.008
TMT-B, seconds^b^	0.860 (4.552)	0.088 (2.364)	.172	.027
Stroop colour	-0.325 (1.478)	0.027 (0.985)	.125	.034
Stroop word	0.112 (1.730)	-0.369 (1.409)	.300	.016
Stroop interference	-0.077 (1.201)	0.071 (0.775)	.470	.008
Reaction time simple, milliseconds^b^	6.706 (11.411)	4.229 (7.995)	.627	.004
Reaction time complex, milliseconds^b^	7.893 (8.229)	8.953 (12.164)	.796	.001

Mean RoC (SD). ANCOVA corrected for age at baseline. *p≤ .05, **p ≤.01. Effect sizes are displayed as partial eta squared  (_p_h^2^) values. Positive scores indicate an improvement over time and negative scores indicate deterioration over time, except for ^b ^where positive scores indicate deterioration and negative scores indicate an improvement. RoC= Rate of Change. UHDRS= Unified Huntington’s Disease Rating Scale. TMS= Total Motor Score. TBS= Total Behavioural Score. MMSE= Mini Mental State Examination, WAIS-R= Wechsler Adult Intelligence Scale-Revised. WMS= Wechsler Memory Scale, SDMT= Symbol Digit Modalities Test (number correct), TMT= Trail Making Test (sec), Reaction time simple= Reaction time single stimulus conditions (milliseconds), Reaction time complex= Reaction time complex conditions (milliseconds).  

*Associations between estimated years to clinical diagnosis and Rates of Change*

Table 5 shows that proximity to estimated clinical diagnosis (EYTD) in carriers was associated with a greater rate of decline on WAIS-R Information (p= .033, r= .397), WMS memory quotient (p= .024, r= .426), WMS concentration (p= .034, r= .396), WMS logical memory (p= .016, r= .444), WMS visual reproduction (p= .012, r= .461) and Reaction time complex condition (p= .046, r= -.410). Excluding the carriers who converted to manifest HD, associations remained between EYTD and rate of decline on WMS memory quotient (p= .045, r= .421), WMS logical memory (p= .028, r= .448) and WMS visual reproduction (p= .005, r= .553).

Table 5. Correlations between estimated years to clinical diagnosis (EYTD) and rate of Change (RoC) in carriers  

**Table:** 

EYTD RoC r-value P		
**Motor assessment** ^ b^		
UHDRS TMS	-.266	.189
Eye movement	-.264	.193
Voluntary movement	-.218	.286
Chorea	-.222	.276
**Behavioural assessment** ^ b^		
UHDRS TBS	-.043	.828
Sadness	-.137	.486
Anxiety	.104	.598
Aggression	-.241	.217
Irritability	.062	.753
**Neuropsychological assessment**		
MMSE, total score	.233	.224
WAIS-R Information	.397	.033*
Digit span	.082	.672
Arithmetic	.338	.073
Picture arrangement	.124	.529
Block design	.267	.170
WMS, memory quotient	.426	.024*
Concentration	.396	.034*
Logical memory	.444	.016*
Visual reproduction	.461	.012*
Associative learning	-.002	.990
Verbal fluency (FAS)	.089	.645
Boston naming test	.130	.501
SDMT, total score	.289	.129
TMT-A, seconds^b^	-.171	.376
TMT-B, seconds^b^	-.328	.082
Stroop colour	.166	.398
Stroop word	.345	.067
Stroop interference	-.022	.910
Reaction time simple, milliseconds^b^	-.214	.315
Reaction time complex, milliseconds^b^	-.410	.046*

Pearson correlation coefficients (r-value) with *p≤ .05. RoC= Rate of Change. Positive scores indicate an improvement over time and negative scores indicate deterioration over time, except for ^b ^where positive scores indicate deterioration and negative scores indicate an improvement. UHDRS= Unified Huntington’s Disease Rating Scale. TMS= Total Motor Score. TBS= Total Behavioural Score. MMSE= Mini Mental State Examination, WAIS-R= Wechsler Adult Intelligence Scale-Revised. WMS= Wechsler Memory Scale, SDMT= Symbol Digit Modalities Test, TMT= Trail Making Test, Reaction time simple= Reaction time single stimulus conditions, Reaction time complex= Reaction time complex conditions. 

**Discussion**

This longitudinal study with a follow-up of seven years demonstrated a significant decline in motor functioning, memory, and concentration in premanifest carriers of the HD gene mutation. Cognitive changes over time could be primarily ascribed to carriers who converted to manifest HD.  

Cross-sectional results at baseline were comparable to previous studies demonstrating abnormalities in carriers in executive function, specifically attention, cognitive flexibility, psychomotor speed,  and inhibitory processes, as assessed with WAIS-R arithmetic, SDMT, TMT and Stroop [5] [6] [34]. Without the carriers who converted to manifest HD during the study, cognitive abnormalities at baseline could still be demonstrated. This indicates that subtle cognitive deviations, especially on executive functions, are present, even long before the onset of HD motor signs and may be related to early deficits in the basal-ganglia circuitry [15] [35]. After seven years additional cross-sectional differences emerged, with carriers showing more motor abnormalities on the UHDRS motor section and worse performance on memory and concentration tasks from the WMS, compared to non-carriers [2] [6] [7] [10] [13].   

    Remarkably, we could not demonstrate a significant decline on the executive tasks that proved sensitive for the earliest cognitive manifestations of HD at baseline. Also, we did not find an association between estimated years to clinical diagnosis and rate of change on these tasks. Practice effects on these type of tasks and familiarity with the test procedures might compensate for subtle cognitive deficits [19]. This is important for the interpretation of longitudinal data. A follow-up study by Paulsen et al. (2001) [36]showed an improvement on executive tasks in individuals at-risk for HD and a decline in converters. This is in accordance with our finding that differences between groups at follow-up, could be ascribed in particular to carriers who converted to manifest HD during the study. Indeed, previous longitudinal studies that did demonstrate a decline on these tasks displayed a much higher rate of carriers converting to manifest HD [20] [21] [36] [37]. Duff et al. (2007) suggest that the amount of individual practice effects in longitudinal studies might provide valuable information on cognitive status and predict long-term cognitive outcome [38]. 

The WMS memory quotient shows an absence of practice effects in carriers compared to non-carriers, suggesting cognitive dysfunction in carriers. Indeed, we did demonstrate a decline in memory function and concentration on the WMS compared to non-carriers. These changes over time could be attributed to the carriers who converted to manifest HD, except for the decline on the concentration subtest. Also, when estimations of age at clinical diagnosis were used, an association with rate of change on the WMS could be demonstrated. Memory decline in individuals approaching clinical disease onset is confirmed by other studies [7] [12] [13]. The fact that we could not detect cross-sectional differences between carriers and non-carriers at baseline on memory tasks is in line with the observation by Snowden at al. (2002) that memory function shows a precipitous decline around the time of clinical onset [20]. Perhaps, concentration changes evolve more slowly and precede the effect on memory tasks. It can be argued, however, that the concentration subtest of the WMS does not reflect selective attention but rather general cognitive slowing, since it is a timed task.   

Interestingly, many longitudinal studies reported mainly on motor and executive tasks, specifically psychomotor speed and cognitive flexibility [21] [36] [39]. This is also reflected in the broadly used UHDRS cognitive section that is highly influenced by motor speed. Our findings confirm the importance of these tasks in detecting premanifest abnormalities. However we advocate the addition of memory tasks for this purpose since memory decline may be a sign of conversion to manifest HD and these tasks have the advantage to lack the motor component.   

Behavioural changes could not be demonstrated in the current study. Complaints about aggression on the UHDRS behavioural section at baseline disappeared in subsequent years and confirm the variability in occurrence of psychiatric symptoms [17]. Furthermore, as many objective, quantitative cognitive tasks are available, these are more prominently represented in the present study design. In current studies, more attention is paid to behavioural and mood changes using extensive batteries of neuropsychiatric questionnaires including the Problem Behaviours Assessment for Huntington Disease (PBA-HD) [40] [8]. Also, in motor functioning more continuous measures of motor function should be included in clinical studies since reliability of the UHDRS motor assessment is somewhat limited, especially when motor signs are very subtle [41]. This is reflected in the large number of non-carriers in our study that are rated with minor soft signs or probable HD. Converters to manifest HD in the current study mainly developed signs of chorea. We suggest that the appearance of subtle choreatic movements is an important specific feature for the motor examiner when diagnosing unquestionable HD. In a recent study, quantitative voluntary neurophysiological motor tasks proved sensitive for subtle motor deficits in carriers more than a decade before estimated clinical onset and might be used more commonly in premanifest HD research [8].   

In the evaluation of longitudinal results of the current and previous studies, many discrepancies appear. Heterogeneity in closeness to clinical disease onset within and between studies and differences in the studied measures are probably the most important factors. Follow-up studies should show international uniformity in inclusion criteria and assessment protocol. Furthermore, longitudinal studies combining clinical and biological measures will provide more insight into the processes underlying clinical changes and may lead to a combination of measures suitable for objectively tracking premanifest HD. Ongoing international, multi-centre, multidisciplinary trials are an important example in improving research on the subject and in realising fast recruitment of study samples with sufficient power. PREDICT-HD [6], TRACK-HD [8] COHORT [42] and REGISTRY [43] are longitudinal observational studies on clinical and biological markers in the evolution of HD. Longitudinal data on these impressive studies will contribute substantially to the knowledge on the phase of onset of HD.   

*Conclusion*

Standardized motor assessments and objective memory and concentration tasks prove sensitive for change, specifically in the phenoconversion phase. Executive tasks were found to be sensitive for subtle cognitive abnormalities in premanifest HD, a decline over time could, however, not be demonstrated on these tasks. Strengths of the current study are the lengthy follow-up and the comprehensive assessment battery, enabling us to detect changes over time. For the purpose of therapeutic trials, however, suitable instruments are still needed to track changes over shorter intervals. Extended follow-up periods appear useful only for clinical purposes since information about clinical disease onset and progression is of crucial importance in improving the psychosocial support for patients and their families. 

    Because of discrepancies between studies and the discontinuous evolvement of clinical changes, uniformity in international research through multi-site collaborative research models will improve premanifest HD research substantially and point to a selection of specific clinical measures sensitive to change in premanifest HD.  

   **Acknowledgements**

None

**Funding information**

None

**Competing interests**

The authors have declared that no competing interests exist. 
